# Fidelity of D.A.R.E. Officers’ Delivery of “keepin’ it REAL” in Elementary & Middle School

**DOI:** 10.1007/s11121-023-01548-8

**Published:** 2023-06-26

**Authors:** Emily R. Beamon, Robert A. Henson, Samantha E. Kelly, William B. Hansen, David L. Wyrick

**Affiliations:** 1 University of North Carolina Greensboro, Greensboro, NC USA; 2 Piedmont Research Strategies, Greensboro, NC USA; 3 Prevention Strategies, Greensboro, NC USA

## Abstract

The goal of the current study is to examine the degree to which measures of quality of implementation and student engagement moderate pretest–posttest changes in mediating variables that are targeted by DARE “keepin’ it REAL.” DARE officers (10 elementary school, five middle school) taught DARE “keepin’ it REAL lessons to 1,017 elementary students (480 boys and 534 girls) and 435 middle school students (217 boys and 215 girls). We examined teachers’ and students’ ratings of elementary and middle schools in response to DARE officers’ delivery of the program. HLM analyses revealed that students’ engagement was a significant and meaningful predictor of changes in targeted mediators. Teachers’ ratings of student responsiveness added little in terms of understanding these outcomes with main effects observed only for students’ ability to respond to bulling and students’ estimates of peer drug use. Teachers’ ratings of the quality of officer implementation, on the other hand, did add to understanding students’ outcomes. Effects were seen for three (peer norms about drug use, decision-making (DM) skills, intentions to avoid drug use) out of six outcome variables and suggest a stronger positive effect for elementary versus middle school students. At least for these three outcomes, understanding quality of implementation added to our ability to interpret results. Specifically, in addition to students’ engagement, quality of implementation (which varied by grade) contributed to achieving positive changes in students’ outcomes.

There is inherent variability in the way in which prevention programs are delivered. Given what might otherwise be a standardized curriculum, implementation will never be perfectly uniform. Teachers delivering a program differ in numerous ways – in their training, age and experience, and broadly speaking, in their backgrounds and expectations. Students also differ in similar ways, as well as in their risk status at the outset of receiving an intervention. In an ideal world, teachers will adapt their teaching style to accommodate individual students’ situations, while delivering a program with maximal fidelity.

The goal of any disseminated prevention program is to provide training and support to teachers so that they can maximize fidelity given whatever classroom circumstances they are in. Interestingly, once a program has been classified as evidence-based, it is rare for fidelity to be systematically assessed. The goal of this study is to document the degree to which DARE officers’ delivery of “keepin’ it REAL” (KIR) achieves high fidelity.

## Quality of Implementation

As with all prevention programs, we expect there to be setting-to-setting, grade-to-grade, and teacher-to-teacher variation in how well DARE might be implemented. Overall, there is extensive evidence that the quality of implementation matters in terms of how effective programs can be at achieving prevention goals (Durlak & DuPre, 2008; Dusenbury et al., 2003). Dane and Schneider (1998) were among the first to differentiate constructs related to fidelity of implementation. They noted that five distinct constructs were possible: (1) adherence, (2) dose, (3) quality of program delivery, (4) participant responsiveness and (5) program differentiation. Durlak and Dupre (2008) expand on this list to include: (6) monitoring of control/comparison conditions, (7) program reach, and (8) adaptation.

Berkel and colleagues (2011) presented an integrated model that describes how program implementation is expected to affect program outcomes. They propose that quality of implementation can be thought of having two basic components: 1) how the intervention is delivered in terms of fidelity, quality, and adaptation; and 2) participant responsiveness, which may be influenced by quality of delivery (including the types of adaptations made). Together, fidelity, quality, adaptation, and participant responsiveness moderate program effectiveness.

Strategies for measuring quality of implementation have included teacher and student self-reports, observations, and ratings of video recordings. Teachers’ self-reports about their own implementation have proven to be inferior to third party assessments (Hansen & McNeal, 1999; Hansen et al., 2014; Lillehoj et al., 2004; Mihalic et al., 2008; Miller‐Day et al., 2013), although self-reports have demonstrated value for some analyses (Hanley et al., 2009; Low et al., 2014). Where multiple methods of assessment are possible and convergence can be achieved, improved accuracy is expected and is recommended.

Hansen and his colleagues (Hansen et al., 1991) were among the first to measure the moderating effect of program “integrity” in prevention research. Since then, a variety of measures of quality of implementation have been developed and reported (Bishop et al., 2014). It has also been assessed examining adherence to the curriculum, teacher engagement (attentiveness, enthusiasm, seriousness, clarity, positivity), and student engagement (attention, participation; Pettigrew, 2015).

## Student Engagement

In addition to the quality of implementation of a program, participant responsiveness, or “student engagement,” (the term preferred in this study), has also been related to a number of student outcomes. It can be classified into four general constructs: academic motivation, school connectedness, caring relations with teachers, and meaningful participation (Scheier & Komrac, 2020). While Scheier and Komrac (2020) found that academic motivation and school connectedness correlated with 7th, 9th, and 11th grade drug use, few studies have explicitly included student engagement as a measure (Durlak & Dupre, 2008; Dusenbury et al., 2003). Observational ratings of student engagement have yielded mixed results. Some studies assessed inter-rater reliability, but not reliability related to student responsiveness (Pettigrew et al., 2013); others found less agreement among raters when examining student engagement (Bishop et al., 2014); and in-class observers rated student involvement differently depending on who delivered the program (Harrington et al., 2001).

Student reports have also been used to assess engagement in programs. Hansen (1996) assessed students’ reactions to both a pilot test of “All Stars” and the implementation of DARE in 7th grade classrooms. Students responded to questions about their experience with the program, the degree to which they would recommend the program to others, and the degree to which they expressed thoughts and feelings during the program. Students were highly engaged with All Stars and experienced more positive outcomes. A recent study of All Stars (Hansen et al., 2019) relied on students’ reports about engagement at posttest. The engagement scale was reliable, and HLM analyses revealed that student engagement at both the student and classroom level significantly moderated pretest–posttest change in targeted mediators.

## DARE Prevention Programs

Initially, the DARE program was delivered during the last year of elementary school (Ennett et al., 2011). In attempt to defray criticism about the ineffectiveness of the original intervention (Rosenbaum & Hanson, 1998), DARE America tested an alternative program in the 2000s (Sloboda et al., 2009). Failing to further find positive outcomes with this alternative intervention, DARE sought out and adopted a program already certified as evidence-based: “keepin’ it REAL.” DARE’s implementation of KIR now includes both elementary (typically 5th grade) and middle school (6th or 7th grade) versions (Day et al., 2017; Hecht et al., 2008; Nordrum, 2014). While the adoption of KIR has provided DARE with an evidence-based program, Caputi and McLellan (2017) have argued that there is not yet evidence that DARE officers’ ability to deliver the program has been demonstrated.

## “keepin’ it REAL”

Prior research on KIR was conducted with regular classroom teachers delivering the middle school intervention (Hecht et al., 2003, 2006). These studies have shown that KIR has the potential to deter the onset of alcohol, cigarette, and marijuana use. A version of the program adapted for fifth grade students did not yield preventive outcomes when results were compared to controls (Hecht et al., 2008).

The elementary and middle school curricula address similar topics, but place different emphasis on and use different methods for addressing these topics. For example, both programs address helping students develop self-efficacy to respond to stress and peer pressure; however, self-efficacy instruction is touched on in six elementary lessons and nine middle school lessons. The specific strategies, Refuse, Explain, Avoid, and Leave (REAL) are taught only in middle school. Both curricula teach and reinforce decision-making skills that are touched on in all ten lessons. Both programs address knowledge and beliefs about the consequences of alcohol, cigarettes, and other drug use (these are included in six elementary and 9 middle school lessons). Bullying is addressed only in the elementary school program and social skills are addressed only in the middle school program. Both programs include brief information designed to correct erroneous perceptions of alcohol and drug use norms.

## DARE Officer Training

During their 80-hour training, DARE officers are trained to ensure and adhere to implementation of curriculum (DARE, 1991). The standard for training DARE officers involves 10 days of instruction (two weeks of consecutive eight-hour, five-day meetings). Instruction focuses on KIR, as well instruction about general pedagogy and logistics. Overall, training includes approximately 29 h of instruction about the elementary program, 11 h of instruction about the middle school program, and six hours of instruction about the high school, K-4, and enhancement modules. Included in training are individual assignments that require officers to use the curriculum guide to develop personalized lesson plans.

Prior to implementing the program with students, each officer trainee views a certified trainer teach lessons and has the opportunity to practice teaching using fellow trainees as a mock classroom of students. Standardization of training has created an international environment in which the wide dissemination of the program can be achieved (Merrill et al., 2006) and likely became helpful with the adoption of KIR in 2013 (Nordrum, 2014). There are several reports in the research literature that support the adoption of KIR (Day et al., 2017; Hecht et al., 2008; Marsiglia et al., 2015, 2019), although not all evaluations produce clear beneficial results have yielded desired outcomes (Elek et al., 2010).

## The Current Study

The goal of the current study is to examine the degree to which measures of quality of implementation and student engagement moderate pretest–posttest changes in mediating variables that are targeted by KIR. We examined teachers’ and students’ ratings within elementary and middle schools in response to DARE officers’ delivery of the program.

## Research Questions

1. Does student engagement in the DARE program predict positive outcomes for students on the six outcome variables across elementary and middle schools?
2. Does achieving high levels of fidelity to the DARE program predict positive outcomes for students across elementary and middle schools?

## Method

### Participants

Table 1 represents the demographic characteristics of both elementary and middle school students. Posttest data was collected from 1,017 elementary school students, across 45 teachers, and ten DARE officers. In contrast, posttest data was collected from 435 middle school students, across eight middle school teachers, and five DARE officers.

**Table 1:** Demographic Characteristics for Elementary and Middle School Students

Variables		Elementary N = 1017	Middle N = 435
Age		Mean = 10.68 S.E.M = .02	Mean = 12.80 S.E.M. = .03
Sex			
	Boy	480 (47.2%)	217 (49.9%)
	Girl	534 (52.5%)	215 (49.4%)
	*Missing*	3 (0.3%)	3 (0.7%)
Race			
	White	633 (62.2%)	242 (55.6%)
	Black / African American	162 (15.9%)	60 (13.8%)
	Hispanic	96 (9.4%)	13 (3%)
	Multiple Races	78 (7.7%)	56 (12.9%)
	Pacific Islander	0 (0%)	49 (11.3%)
	Another Race	25 (2.5%)	8 (1.8%)
	Asian	13 (1.3%)	1 (0.2%)
	Native American	0 (0%)	3 (0.7%)
	*Missing*	3 (0.3%)	3 (0.7%)
Hispanic	Yes (Hispanic/Latino)	148 (14.6%)	85 (19.5%)
	No (Not Hispanic/Latino)	849 (83.5%)	335 (77%)
	*Missing*	20 (2%)	15 (3.5%)
Who do you live with most of the time?			
	Two parents	821 (80.7%)	308 (70.8%)
	Mother only	144 (14.2%)	90 (20.7%)
	Father only	16 (1.6%)	18 (4.1%)
	Someone else	32 (3.1%)	14 (3.2%)
	*Missing*	4 (0.4%)	5 (1.1%)

## Measures

Two sets of measures were collected, *teacher ratings about fidelity and engagement* and *student self-reports about engagement*. For each DARE KIR lesson, teachers were supplied with a one-page form on which to make ratings. Both elementary and middle school KIR have 10 lessons. For each lesson, teachers noted 1) which lesson activities were completed (yes/no), 2) what percent of students within the class had lesson objectives had been achieved (70% or less; 80%; 90%; or 100%), 3) how energetic and 4) how prepared the officer was, 5) how attentive students were, 6) how many students were engaged in the lesson, 7) answered questions, 8) asked questions, and 9) how many student discipline problems occurred (all items were coded on a 0–10 point scale and were averaged).^1^ For the purposes of the analyses, there were two teacher-rated fidelity ratings: how responsive the students were to the lessons and how responsive the DARE officer was to the students.

### Student Self-Reports about Engagement

At posttest, the student completed questions about their participation with KIR, including: 1) Did the DARE officer do a good job teaching DARE? 2) Did you enjoy DARE? 3) How often did you share your opinion during DARE? 4) How often did you pay attention during DARE? 5) Did DARE help you think about what was important to you as you grow older? 6) Did the DARE officer listen when you spoke in class? 7) Does your DARE officer know your name? and 8) Did you like your DARE officer? Student engagement items were scored from 0–10, averaged across all six posttest ratings completed by students (α = .788).

### Student Outcome Variables

#### Attitudes toward Police

Students were asked 7 items rating law enforcement officers in terms of if they were viewed as being helpful, trustworthy, friendly, fair, and respected. There was good reliability at pretest for elementary (α = .861) and middle school (α = .921) students.

#### Bullying Response Skills

  Elementary and middle school students responded to three prompts about bullying that reflected their skill to respond should they observe bullying occurring. At pretest, elementary students (α = .602) and middle school students (α = .634) responses reflected adequate internal consistency.

#### Decide Skills

Students in both grades responded to five items assessing thinking about choices, acting without thinking, assessing potential health consequences, comparing good and bad outcomes, and assessing the future implications of their choice. Internal consistency at pretest was good for both elementary (α = .753) and middle school (α = .810) students.

#### Intention to Avoid Drug Use

  Elementary students responded to three prompts about future intentions; being willing to sign a pledge to not drink alcohol, living a drug-free life, and telling someone they do not plan to smoke (α = .565). Middle school students were provided with an additional four prompts that included alcohol, cigarette, and marijuana items (α = .769).

#### Communication with Parents

Both elementary and middle school students responded to six items about drinking alcohol, smoking cigarettes, peer pressure, being bullied, general responsibilities, and discussions about topics of importance to the student. At pretest both elementary (α = .753) and middle school (α = .787) scales had acceptable internal consistency.

#### Beliefs about Peer Drug Use Norms

Elementary students answered four normative belief items. Two about descriptive norms, and two assessed injunctive norms. Internal consistency at pretest for elementary students was modest (α = .578). Middle school students answered an additional two items about marijuana descriptive/injunctive norms (α = .703).

## Analysis Plan

### Factor Analysis and Internal Consistency of Teacher Ratings

The data was analyzed to create scales useful for assessing concordance between teacher and student constructs. A principal component factor analyses was completed using the teachers’ fidelity ratings and students’ self-reports about engagement. Each analysis used Varimax rotations and included all measured variables with factors created when eigenvalues ≥ 1. Items that loaded on resulting factors were analyzed for internal consistency using Cronbach’s alpha. Using scaled data, aggregated at the DARE officer level, we completed correlations, examining the concordance between teachers’ and students’ ratings.

### Hierarchical Linear Modeling (HLM)

In this particular case students are naturally clustered within classes. Thus, it is possible that any two students within a given class are more similar than two students in different classes. In addition, sets of teachers share the same DARE officer. Similar to students, teachers whose classes receive the program from the same DARE officer may be more similar than teachers who have different DARE officers. Nested data, such as this, have the potential of violating the independence assumption of a traditional linear regression model.

To answer our specific research question while also addressing specific concerns of dependent observations, Hierarchical Linear Models where used (Raudenbush & Bryk, 2002). HLM (also commonly known as mixed models) can be used when data is naturally clustered based on different levels. In this case, students (level 1) are nested within teachers (level 2) and teachers are nested within DARE officers (level 3). Each level, is used to describe variability within the level “above it.” For example, a linear model is used to describe differences between students who are nested within teachers. That relationship is then modeled as a function of teacher level variables, which in turn is allowed to vary across DARE officers. The variability that is defined at each level within the HLM analysis is what models dependencies within the data due to this nesting. Next, the models at each level that are used to answer the research questions are presented.

Level 1 (student) is used to model the relationship between the dependent variable and student engagement while controlling for the pretest. Specifically, the model is defined as:

1 $${\mathrm Y}_{\mathrm{ijk}}\hspace{0.17em}\;=\;\hspace{0.17em}{\mathrm{Pi}}_{0\mathrm{jk}}\hspace{0.17em}\;+\;\hspace{0.17em}{\mathrm{Pi}}_{1\mathrm{jk}}\;(\mathrm{pretest})\;\hspace{0.17em}+\;\hspace{0.17em}{\mathrm{Pi}}_{2\mathrm{jk}}\;(\mathrm s\mathrm t\mathrm u\mathrm d\mathrm e\mathrm n\mathrm t\;\mathrm e\mathrm n\mathrm g\mathrm a\mathrm g\mathrm e\mathrm m\mathrm e\mathrm n\mathrm t)\hspace{0.17em}+\hspace{0.17em}{\mathrm e}_{\mathrm{ijk}}$$

where Pi_0jk_ describes the average posttest after adjusting (or controlling) for pretest and student engagement, Pi_1jk_ is the grand mean centered pretest score for each student, and Pi_2jk_ is the grand mean centered student engagement scores as rated by the students.

As level 2 (classroom) specific variables are used to model how the student relationships are affected by teacher-level variables. Because this relationship is defined by the coefficients in the level 1 (student) model, it is possible to define a level 2 (classroom) model such that there is an equation modeling each of the three level 1 coefficients (Pi_0jk_, Pi_1jk,_ and Pi_2jk_) each of which is modeled as follows:

$$\mathrm{Equation\;1: Level}\;1:\;{\mathrm{Pi}}_{0\mathrm{jk}}\hspace{0.17em}\;=\;\hspace{0.17em}{\mathrm B}_{00\mathrm k}\hspace{0.17em}\;+\;\hspace{0.17em}{\mathrm B}_{01\mathrm k}\;(\mathrm{Teacher}\;\mathrm{Fidelity})\hspace{0.17em}\;+\;\hspace{0.17em}{\mathrm B}_{02\mathrm k}\;(\mathrm{Grade})\hspace{0.17em}\;+\;\hspace{0.17em}{\mathrm B}_{03\mathrm k}\;(\mathrm{Grade}\;x\;\mathrm{Teacher}\;\mathrm{Fidelity})\hspace{0.17em}\;+\;\hspace{0.17em}{\mathrm r}_{0\mathrm{jk}}$$

$$\mathrm{Equation\;2: Level}\;2:\;{\mathrm{Pi}}_{1\mathrm{jk}}\hspace{0.17em}\;=\;\hspace{0.17em}{\mathrm B}_{10\mathrm k}$$

$$\mathrm{Equation\;3:\;Level}\;3:\;{\mathrm{Pi}}_{2\mathrm{jk}}\hspace{0.17em}\;=\;\hspace{0.17em}{\mathrm B}_{20\mathrm k}\hspace{0.17em}\;+\;\hspace{0.17em}{\mathrm B}_{21\mathrm k}\;(\mathrm{Teacher}\;\mathrm{Fidelity})\hspace{0.17em}\;+\;\hspace{0.17em}{\mathrm B}_{22\mathrm k}\;(\mathrm{Grade})\hspace{0.17em}\;+\;\hspace{0.17em}{\mathrm B}_{23\mathrm k}\;(\mathrm{Grade}\;x\;\mathrm{Teacher}\;\mathrm{Fidelity})\hspace{0.17em}\;+\;\hspace{0.17em}{\mathrm r}_{2\mathrm{jk}}$$

where B_01k_ and B_21k_ describe the effects of the group mean centered ratings of teacher fidelity (depending on subsequent analyses, this is defined as either teacher ratings of student engagement with DARE, or teacher ratings of DARE officer engagement with the classroom). B_02k_ is difference between students because it is the effect when moving from grade 0 to grade 1 (0 = elementary, 1 = middle), and B_03k_ is the interaction of teacher fidelity ratings by grade.

Finally, this relationship could depend on characteristics of each DARE officer, which is defined by the level 3 (D.A.R.E. Officer) model. In this case, there are no specific variables for a DARE officer. However, it is possible that there are systematic differences between DARE officers such that the dependent variable is higher for some DARE officers when compared to others. As a result, level three models this variation for the intercept and explicitly assuming that all other relationships are the same across DARE officers. The intercept (as seen in Eq. 1) explicitly assumes that﻿:

2 $${\mathrm{B}}_{00\mathrm{k}}\hspace{0.17em}=\hspace{0.17em}{\mathrm{Gamma}}_{000}\hspace{0.17em}+\hspace{0.17em}{\mathrm{u}}_{00\mathrm{k}} (\mathrm{random\;effect})$$

3 $${\mathrm{B}}_{\mathrm{pqk}}\hspace{0.17em}=\hspace{0.17em}{\mathrm{Gamma}}_{\mathrm{pq}0}$$

where Gamma_000_ describes the effect of posttest differences between DARE officers and that these differences have been allowed to vary between officers (u_00k_).

HLM is a powerful approach that allows the effects of the independent variables to be explored and to test for possible moderation of grade, student engagement, and ratings of teacher fidelity while also accounting for possible dependencies in the data that would have otherwise been ignored. Analyses were run separately in SPSS for the six individual dependent variables (attitudes towards police, bully response skill, DM skill, intention to avoid drug use, parent communication, and peer norm drug use). Due to low reliability coefficients, we excluded Beliefs about Drugs and Refusal Skills from these analyses.

## Results

Elementary teachers contributed ratings for 425 lessons. Middle school teachers contributed ratings for 50 lessons. We calculated the rates at which elementary and middle school teachers returned ratings based on the total numbers of forms teachers completed. On average, elementary school teachers returned 94.4% of fidelity forms with 80.0% of teachers returning all ten forms. Middle school teachers returned 62.5% of fidelity assessment forms with only 25.0% of teachers returning all ten forms.

Three factors of fidelity emerged: *teacher rating of student responsiveness* (TRSR; four items; α = .688), *teacher rating of the officer’s quality of implementation* (TROQI; four items; α = .553), and *teacher rating of activities completed* (TRAC). Comparing the factor analysis outcomes to prior conceptualizations of fidelity by Dane and Schneider’s (1998) classification, Factor 1 (TRSR) reflects an assessment of participant responsiveness from the teacher’s perspective; items included teacher ratings of students asking and answering questions, the percentage of students engaged, and number of student objectives completed. Factor 2 (TROQI) corresponds to quality of program delivery, also from the teacher’s perspective; items included teacher ratings of the officer being prepared and energetic, officers holding students’ attention, and the officer properly dealing with discipline problems. Factor 3 (TRAC) corresponds to adherence rated by the teacher. The student engagement scale reflects an alternative assessment for participant responsiveness. Due to the regimented manner of the DARE curriculum, data was highly skewed, as almost all activities were completed by the officers. For the purposes of analyses, we will be examining TRSR and TROQI. Only one factor emerged when we examined student ratings of engagement, so they will be analyzed as a whole.

## Correlations

Correlations among the two teacher ratings and one student measure were calculated. With only 15 officers, correlations needed to be large for significance to be observed. Only two correlations emerged as being statistically significant: *TROQI* with *student’s ratings of engagement* (*r* = .81; *p* < .0001) and *TROQI* with *TRSR* (*r* = .70; *p* = .004). it is the former correlation that has the greatest importance for interpreting intervention outcomes. The strength of correlation rested on a concordance of judgments by teachers and students. This was primarily influenced by ratings of two DARE officers who delivered the program to middle school students and who, based on both teacher and student ratings, “performed poorly.” Elementary teachers and students both gave high average ratings (9.40 for TROQI and 9.32 for *student engagement*). On the other hand, overall average teacher and student ratings for middle school DARE delivery was 8.81 and 7.00, respectively. These differences were significant for both teacher ratings (*t* (464) = 3.50, *p* = .001) and student ratings (*t* (1,591) = 24.32, *p* < .0001).

## HLM Results

Prior to running any models, an unconditional model was run so that it is possible to determine the proportion of variance attributed to each level (Levels 1, 2, and 3) of each outcome variable at Levels 1, 2, and 3. If students were missing data at L3 or L2, data was dropped from analyses as this information was integral to the research question. Only students with both pretest and posttests were included in analyses. Table 2 is the intraclass correlation coefficient- the proportion of variance attributed to within classes, between classes within DARE Officer, and between DARE Officers. Particularly interesting is the attitudes towards police and peer norm drug use, which have highest percent of variance attributed to officers, in contrast to the other outcomes.

**Table 2:** Intraclass Correlation Coefficients

	Attitudes toward Police	Bully Response Skill	Decision Making Skill	Intention to Avoid Drug Use	Parent Comm-unication	Peer Norm Drug Use
Within Class	.81	.91	.98	.93	.95	.78
Between Class	.05	.01	.00	.02	.02	.06
Between DO	.14	.08	.02	.05	.03	.16

*DO* DARE Officer

### Student Engagement x Teacher Ratings of Student Responsiveness

The first set of HLM analyses included *student’s ratings of their engagement* (SE) and *teacher’s ratings of student responsiveness* to the DARE officer (TRSR). Level 1 HLM models examined how individual-level characteristics were associated with each of the six outcome variables (see Table 3). The intercept represented the adjusted average values of the dependent variables after controlling for pretest scores on each respective outcome variable (represented in Eq. 1). Table 3 demonstrates that TRSR predicted bully response (B_01k_ = -.10, p < .05) and peer norm drug use (B_01k_ = -.12, p < .05). However, it was not related to attitudes towards the police, DM skill, intention to avoid drug use, or parent communication. It is important to note that while parent communication had no significant main effect with respect to TRSR and grade, the interaction of grade x TRSR was significant (B_03k_ = -.28, p < .05).

**Table 3:**
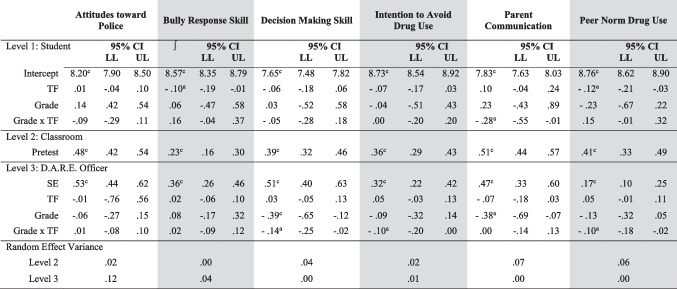
HLM Model of Student Engagement and Teacher Ratings of Student Responsiveness (TRSR﻿)

*CI* Confidence Interval, *LL* lower limit, *UL* upper limit, *TF* Teacher Fidelity (TRSR), *SE * Student Engagement

Y_ijk_ = Pi_0jk_ + Pi_1jk_ (pretest) + Pi_2jk_ (Student Engagement) + e_ijk_

Equation 1: Level 1 (Student): Pi_0jk_ = B_00k_ + B_01k_ (Teacher Fidelity) + B_02k_ (Grade) + B_03k_ (Grade x Teacher Fidelity) + r_0jk_

Equation 2: Level 2 (Classroom): Pi_1jk_ = B_10k_ (Pretest)

Equation 3: Level 3 (D.A.R.E. Officer): Pi_2jk_ = B_20k_ (Student Engagement) + B_21k_ (Teacher Fidelity) + B_22k_ (Grade) + B_23k_ (Grade x Teacher Fidelity) + r_2jk_

^*^p < .05; ^**^p < .01; ^***^p < .001

There were significant, strong main effects for pretest scores (Eq. 2). While these effects were all significant, we were controlling for pretest, so it was to be expected. Across the outcome variables, the effect is reasonably consistent, with the lowest effect for bully response skill (B_10k_ = .23; p < .001), and the highest for parent communication (B_10k =_.51; p < .001).

Equation 3 tests for moderating effect of TRSR, grade, or their interaction with respect to the effect of SE on each of the six outcome variables. Grade was significant among DM (B_22k_ = -.39, p < .001), and parent communication (B_22k_ = -.38, p < .05). While there are two main effects among our outcome variables, there is an interaction between grade and TRSR. When this interaction is significant, both variables may be important, even though one or both their main effects is not significant. This is the case in predicting DM skills, intention to avoid drug use, and peer norm skills where the interaction is significant with respect to DM (B_23k_ = -.14, p < .05), intent (B_23k_ = -.10, p < .05), and peer norm use (B_23k_ = -.10, p < .05).

**Fig. 1:**
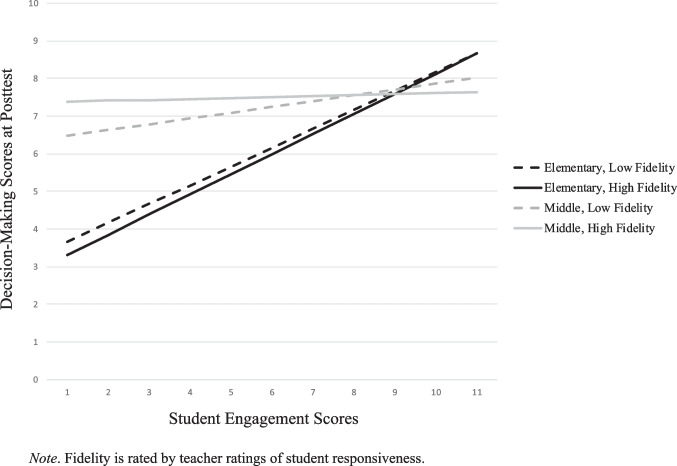
Student Engagement x Teacher Ratings of Student Responsiveness for Decision-Making Skills

Figures 1 through 4 depict three-way interactions for both fidelity measures, and present data for elementary (blue) and middle school (red), with low (dotted) and high (solid) levels of fidelity. Figure 1 depicts the three-way interaction for *grade*, *SE*, and *TRSR* in the classroom on *DM skills*. The moderating effect for grade on student engagement depended on the fidelity ratings, such that the relationship of student engagement on decision-making skills, for example, dissipated when moving from elementary to middle school. The relationship between SE and DM skills nearly disappeared in middle school, and even more so in cases of high fidelity.

**Fig. 2:**
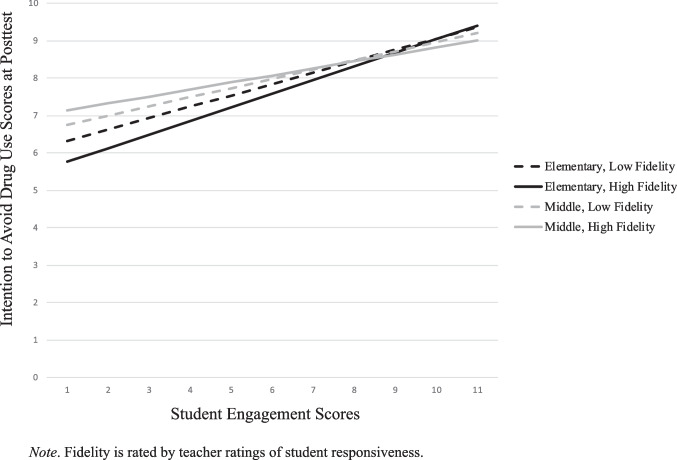
Student Engagement x Teacher Ratings of Student Responsiveness for Intention to Avoid Drug Use

Similar to *DM skills*, there was a general, positive association in students’ engagement and ratings of *intentions to avoid drug use* at posttest (Fig. 2). The relationship of SE in predicting intention to avoid drug use only significantly decreases in cases of middle with high fidelity. When elementary school students were in a classroom that was perceived by the teacher as highly responsive, it was most detrimental to the students to not be engaged (lower intentions to avoid drug use) Among middle school students, there was a similar positive association. Teachers who rated the classrooms as either low or high on student responsiveness saw similar effects at posttest (with high *responsiveness* predicting greater *intentions to avoid drug use*). However, once student engagement ratings increased, higher levels of student engagement for low responsive classrooms were associated with greater *intentions to avoid drug use*.

### Student Engagement x Teacher Ratings of Officers’ Quality of Implementation (TROQI)

The second set of analyses included *student’s ratings of their engagement* (SE) and *teacher’s ratings of officer implementation* to the classroom as the measure of fidelity. There were significant main effects for both pretest scores and student ratings of student engagement for all six outcome variables (see Table 4). Similar to the first set of analyses, the intercept represented the adjusted average values of the dependent variables after controlling for pretest scores on each respective outcome variable (Eq. 1). Similar to TRSR, there were significant, strong main effects for pretests scores (Eq. 2; B_10k_) ranging from 0.23 (bully response skill; p < .001) to 0.51 (parent communication; p < .001).

**Table 4:**
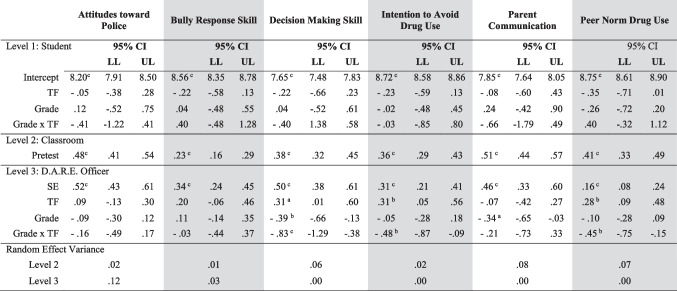
HLM Model of Student Engagement and Teacher Ratings of Officer Quality of Implementation (TROQI﻿)

*CI* Confidence Interval, *LL* lower limit, *UL* upper limit, *TF* Teacher Fidelity (TROQI), *SE * Student Engagement﻿

Y_ijk_ = Pi_0jk_ + Pi_1jk_ (pretest) + Pi_2jk_ (Student Engagement) + e_ijk_

Equation 1: Level 1 (Student): Pi_0jk_ = B_00k_ + B_01k_ (Teacher Fidelity) + B_02k_ (Grade) + B_03k_ (Grade x Teacher Fidelity) + r_0jk_;

Equation 2: Level 2 (Classroom): Pi_1jk_ = B_10k_ (Pretest)

Equation 3: Level 3 (D.A.R.E. Officer): Pi_2jk_ = B_20k_ (Student Engagement) + B_21k_ (Teacher Fidelity) + B_22k_ (Grade) + B_23k_ (Grade x Teacher Fidelity) + r_2jk_

^*^p < .05; ^**^p < .01; ^***^p < .001

In this analysis, Eq. 3 was testing the moderating effect of TROQI, grade, and their interaction, with respect to the effect of student engagement, on each of the six outcome variables. There were significant main effects for TROQI on DM skills (B_21k_ = .31; p < .05), intention to avoid drug use (B_21k_ = .31; p < .01), and peer norm drug use (B_21k_ = .28; p < .01). This can be interpreted as, for the average elementary teacher rating of officer implementation, DM skills increased 0.50 for every one unit increase in SE and an additional 0.31 unit increase as officer implementation ratings increased by one. However, the significant interaction suggests that as officer implementation ratings increased, and grade increased to middle school students, the effect decreased 0.83. This effect was similar for both intentions to avoid drug use and peer norms.

**Fig. 3:**
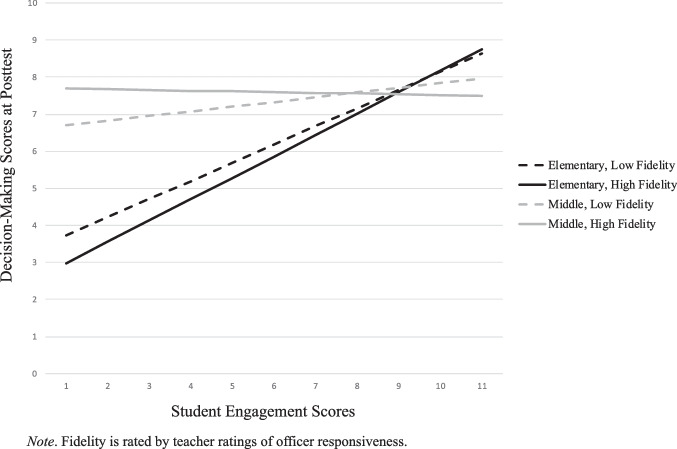
Student Engagement x Teacher Rating Of The Officer’s Quality Of Implementation for Decision-Making Skills

**Fig. 4:**
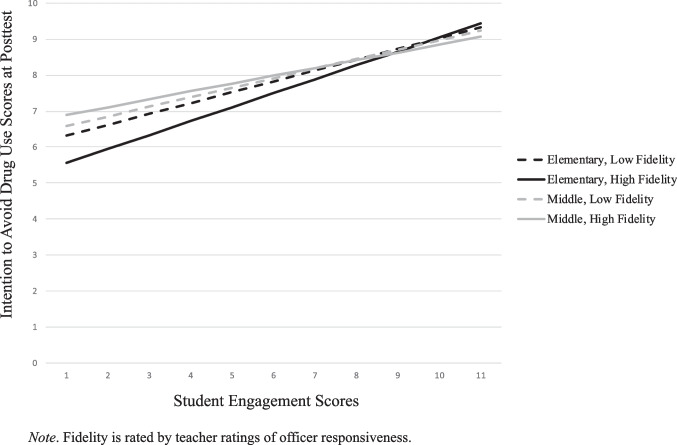
Student Engagement x Teacher Rating Of The Officer’s Quality Of Implementation for Intention to Avoid Drug Use

Figures 3 & 4 present the outcome scores for DM skills and intention to avoid drug use, respectively, considering student engagement, TRQOI, and grade. When elementary school teachers rated their classrooms as either low or high officer implementation, student engagement was significantly predictive of decision-making skills at posttest (Fig. 3). However, for middle school students, when officer implementation was high, there was a negative association with decision-making at posttest and student engagement. The opposite was true for when teachers rated the officer as low on implementation. For students who had a DARE officer who was rated low on implementation, student engagement was more predictive of positive change at posttest.

Regarding intention to avoid drug use, if the officer was rated as *not* providing a high level of implementation, student engagement predicted change at posttest, but to a lesser extent (Fig. 4). Middle school students had a similar effect. When middle school teachers rated the DARE officer as implementing with a high level of implementation, students had the highest *intentions to avoid drug use*, until student engagement was high. At very high levels of student engagement, posttest *intentions to avoid drug use* was at its highest when officer quality of implementation was low.

## Discussion

DARE America adopted an elementary and middle school version of “keepin’ it REAL.” In order to achieve high levels of fidelity, DARE officers receive a minimum of 80 hours of training. Our analyses examined the degree to which there was a fidelity payoff for this training as observed by multiple measures of quality of implementation. It is likely that we had significant findings for some of our mediators (DM skills, intention to avoid drug use, and peer norms) and not others, as these are actively emphasized in the DARE curriculum. If DARE aims to target additional mediators, they may benefit from revisions to the curriculum that purposefully highlight attitudes towards the police, bully response, and parent communication.

Students’ ratings of engagement and teachers’ ratings of quality of implementation were highly correlated. As judged by students’ ratings of engagement and teachers’ ratings of implementation support a high fidelity for the elementary program. In contrast, there were lower ratings by students and teachers for the quality of middle school implementation. Middle school students were less engaged and teachers viewed officers’ as less energetic, achieving less student attentiveness, and having more student discipline problems. There is likely an age effect that is in play with the delivery of KIR by DARE officers. Elementary students are more likely to be engaged with the program simply due to their developmental stage. Middle school students are beginning to enter puberty, gain increased independence, and are increasingly likely to engage in anti-social behaviors and to feel less attached to school the older they get (Oelsner et al., 2011; Simons-Morton et al., 1999). It is important for officers to continue to promote engagement with the program, with special attention paid to middle school students.

HLM analyses revealed that students’ engagement was a significant and meaningful predictor of change in targeted mediators. Student engagement was an important factor when predicting outcome variables at posttest, but more so among elementary school students. Individually, teachers’ ratings of student responsiveness added little in terms of understanding these. We interpret this to mean that students were more insightful about their reactions to, and engagement with the program than were teachers. Consistent with Hansen et al. (2019), student engagement continues to make a considerable contribution in predicting positive change.

In contrast, there were significant main effects when examining teachers’ ratings of the quality of officer implementation when understanding students’ outcomes. As student engagement increased, and as officers were rated more responsive and engaged with students in the classroom, there were more positive changes in student scores. This suggests that, when student engagement is low, officer implementation is highly predictive of changes in the outcome variable. The program is dependent on the DARE officer to be charismatic and engaging, and interact with the students in a way that can counteract students with low engagement scores. However, among the older, middle school students, high implementation ratings did not appear have a significant effect on the outcome variables. If the officer was unable to capture the students’ attention and teach the material well, it didn’t necessarily matter how engaged the student was, they likely would not have any change in their outcome.

There were a number of limitations in this current study. Due to low reliability of two of the initial eight constructs, we decided to exclude both Beliefs about Drugs and Refusal Skills from our analyses. Future studies should consider expanding our operational definition of both constructs to improve its overall measurement. As the nature of this study was to examine the actual implementation of DARE curriculum to elementary and middle school students, and how responsive officers were in the classroom, we are inherently lacking a control group. While there are limitations in the ability to make causal associations without having a control group, our pre-post design was able to reveal significant associations among fidelity, grade, and student responsiveness. Future studies should continue exploring these constructs with a full, randomized controlled trial design to examine causation.

## Conclusion

DARE America utilizes “keepin’ it REAL” as an intervention for drug and alcohol use among elementary and middle school students. DARE officers are required to undergo extensive and thorough training to understand the curriculum, learn how to teach it, but most notably learn how to be engaging with students. In some respects, it appears that this latter piece is what may be most integral to targeting mediators- especially among elementary students. DARE America may continue to conceptualize ways to keep middle school students engaged, while achieving quality of implementation.

## Data Availability

The datasets generated by the survey research during and/or analyzed during the current study are available in the Harvard Dataverse repository, 10.7910/DVN/RM8PRV.

## Footnotes

1 Coding responses from 0 to 10 allows scores from multiple items to be combined with equal weights.
